# The Incidence of Depression among female patients with Schizophrenia Hospitalized in the Clinical Hospital of Psychiatry, Chișinău, R. of Moldova

**DOI:** 10.1192/j.eurpsy.2025.1216

**Published:** 2025-08-26

**Authors:** V. Țapeș, I. Cosciug, I. Deliv, L. Sanduleac

**Affiliations:** 1 Mental Health, Clinical Psychology and Psychotherapy Department, University of Medicine and Pharmacy “Nicolae Testemitanu”, Chisinau, Moldova, Republic of

## Abstract

**Introduction:**

In schizophrenia, depressive and negative symptoms often overlap, complicating the diagnosis. Symptoms such as lack of energy, anhedonia and association make the distinction difficult. A subjective state of sadness may indicate depression, while affective flattening is characteristic of schizophrenia. Other symptoms relevant to diagnosing depression include hopelessness, negative self-assessment, guilt, anxiety, and self-destructive thoughts.

**Objectives:**

This study, conducted over 6 months, aims to establish both the rate and the total number of patients with schizophrenia, suffering from depression to provide a better understanding of the clinical particularities of this type of comorbidity.

**Methods:**

The research provided for a cross-sectional study to assess the incidence of depression among patients with schizophrenia hospitalized in the Clinical Psychiatric Hospital of Chisinau during the reference period. The Calgary depression scale for schizophrenia (CDSS) has been used as an evaluation tool to highlight the presence of depressive symptoms, while PANSS (negative symptoms) has been used, to measure their severity in schizophrenia. The data collection process involved structured questionnaires, semi-structured interviews with patients and clinical history analysis to obtain additional information.

**Results:**

The study included 155 women diagnosed with schizophrenia, aged between 18 and 55. Of these, 28.39% had more than two admissions during the year. Most of the patients included in the study (73.55%) suffered from F20.0, the rates of other forms of schizophrenia being: F20.1 (1.29%); F20.2 (14.84%); F20.3 (9.68%) and F20.9 (0.65%). According to the questionnaire applied to patients with different types of schizophrenia, it was observed that, 40.65% achieved a total score of more than 6 points on the CDSS scale (clinically significant depression) and 34.92% required repeated hospitalisation in the same year. Of those repeatedly admitted, 74.6% suffered from F20.0; 14.29% (F20.2) and 11.11% (F20.3), which probably could be a confirmation of the increased severity of the patient’s condition in case of comorbidity – schizophrenia-depression.

**Conclusions:**

It can be assumed that the comorbidity of schizophrenia-depression negatively influences the social recovery process, but also the quality of life of patients; increases the risk of relapse. Patients with depression often experience higher rates of hospitalization because depressive symptoms can lead to emotional instability, cognitive impairments, undermining interpersonal relationships, social networking and reintegration. It is essential that mental health professionals identify and treat depression in patients with schizophrenia in order to increase their quality of life.

**Disclosure of Interest:**

None Declared

